# Bioactive Glass-Ceramic Foam Scaffolds from ‘Inorganic Gel Casting’ and Sinter-Crystallization

**DOI:** 10.3390/ma11030349

**Published:** 2018-02-27

**Authors:** Hamada Elsayed, Acacio Rincón Romero, Giulia Molino, Chiara Vitale Brovarone, Enrico Bernardo

**Affiliations:** 1 Department of Industrial Engineering, University of Padova, Via Marzolo 9, 35131 Padova, Italy; hamada.elsayed@unipd.it (H.E.); acacio.rinconromero@unipd.it (A.R.R.); 2 Ceramics Department, National Research Centre, El-Bohous Street, Cairo 12622, Egypt; 3 Dipartimento Scienza Applicata e Tecnologia, Politecnico di Torino, 10129 Torino, Italy; giulia.molino@polito.it (G.M.); chiara.vitale@polito.it (C.V.B.)

**Keywords:** CEL2 glass, glass-ceramics, alkali activation, gel casting

## Abstract

Highly porous bioactive glass-ceramic scaffolds were effectively fabricated by an inorganic gel casting technique, based on alkali activation and gelification, followed by viscous flow sintering. Glass powders, already known to yield a bioactive sintered glass-ceramic (CEL2) were dispersed in an alkaline solution, with partial dissolution of glass powders. The obtained glass suspensions underwent progressive hardening, by curing at low temperature (40 °C), owing to the formation of a C–S–H (calcium silicate hydrate) gel. As successful direct foaming was achieved by vigorous mechanical stirring of gelified suspensions, comprising also a surfactant. The developed cellular structures were later heat-treated at 900–1000 °C, to form CEL2 glass-ceramic foams, featuring an abundant total porosity (from 60% to 80%) and well-interconnected macro- and micro-sized cells. The developed foams possessed a compressive strength from 2.5 to 5 MPa, which is in the range of human trabecular bone strength. Therefore, CEL2 glass-ceramics can be proposed for bone substitutions.

## 1. Introduction

Owing to the worldwide increase of population and raise of life expectancy, there is an increasing demand for bone grafts or synthetic materials that can potentially replace, repair or regenerate bone defects [1]. Tissue engineering (TE) is one of the fundamental solutions, implemented to challenge this problem [2]. Natural and synthetic materials, which are used to supply, replenish or enhance the living tissue functionality, are regarded as ‘biomaterials’ [3]. Within them, ‘bioceramics’, to be used for repairing and reconstructing of diseased or damaged parts of the musculo-skeletal system, - also known as ‘bioceramics’, are classified as bioinert (alumina, zirconia), bioresorbable (tricalcium phosphate) or even bioactive (hydroxyapatite, bioactive glasses, and glass-ceramics) [4]. A highly porous structure is often appreciated, e.g., for tissue ingrowth [5].

The bioactivity attributed to some glasses is due to the formation of a hydroxyl carbonated apatite layer (HCA) on their surface similar to bone mineral [5]. This HCA layer forms as a result of a rapid sequence of chemical reactions on the surface of the implant when in contact with body fluids. A well-recognized method to estimate the bone-bonding potential ability of a material, according to Kokubo et al. [6], involves its immersion into simulated body fluid (SBF), followed by the evaluation of bone-like apatite formation on the surface. In other words, the behavior in vivo may be predicted by using the SBF method in vitro, and observing the occurrence of several reactions, including the rapid release of soluble ionic species and the formation of a high surface area layer, consisting of hydrated silica and polycrystalline carbonated hydroxyapatite (HCA) [7,8], under rather strict conditions of pH. In fact, it is known that osteoblasts prefer a slightly alkaline medium (pH = 7.8) [9]; severe changes in pH, as a result of ion release, can inhibit osteoblast activity and can cause cell necrosis or apoptosis [10,11].

CEL2 bioactive glass, belonging to the SiO_2_–P_2_O_5_–CaO–MgO–K_2_O–Na_2_O system, was specifically tailored to control pH variations, due to ion leaching phenomena, when in contact with physiological fluids. Therefore, CEL2 glass features a lower overall alkali content (less than 20 mol %) and a slightly higher P_2_O_5_ content (3 mol %) compared to commercial bioactive glasses. CEL2 glass-ceramic was effectively found to be highly biocompatible and bioactive; in addition, unlike in many bioactive glasses, the positive effect on the mechanical properties imparted by the partial crystallization is not negatively counterbalanced by any decrease of its bioactivity [12,13].

As mentioned above, many bioceramics are in form of highly porous, open-celled bodies, also known as ‘scaffolds’. Glass and glass-ceramic scaffolds can be prepared by using different methods, such as free form fabrication techniques [14], sponge replication [15,16], starch consolidation [17] and burn-out of sacrificial polymeric particles [18]. Concerning CEL2 glass-ceramic, macroporous scaffolds for possible use as bone substitutes were prepared both by sponge replication [13] and burn-out method [19]. Both methods imply the quite delicate removal, by thermal degradation, of an organic phase, acting as a template for the cellular structure (e.g., operating with polyurethane sponge) or for the pores (e.g., operating with fugitive pore formers), followed by viscous flow sintering of glass powders, in turn accompanied by a partial crystallization. Especially in the case of sponge replication, the obtainment of high quality components relies on a careful selection of all processing conditions. Before thermal processing, the impregnation of the starting sponge, with an aqueous slurry of glass powders added with a binder (polyvinyl alcohol, PVA), must be absolutely uniform. Upon firing, the viscous flow of the glass should be enough to fill the cavities left by the burn-out of former polymeric struts otherwise low mechanical strength can be expected [20].

The present paper illustrates the extension to CEL2 glass-ceramics of a gel casting process, recently established [21,22]. The method implies the obtainment of cellular structures by direct foaming of engineered glass slurries, followed by sintering. More precisely, CEL2 glass powders were exposed to an alkaline solution, with partial dissolution. The gelation of partially dissolved products (‘inorganic gel casting’) caused a marked hardening of glass suspensions, so that they could be significantly foamed by air incorporation, under intensive mechanical stirring, with the help of a surfactant. Highly porous cellular structures could be tuned simply by adjusting the formulation of slurries; the crystallization of CEL2, upon firing, was significant in ‘freezing’ the microstructure, impeding any viscous collapse.

## 2. Experimental Procedure

### 2.1. Starting Glass

The chosen bioactive glass used as a reference material for the present investigation consisted of a glass belonging to the SiO_2_–P_2_O_5_–CaO–MgO–Na_2_O–K_2_O system. This reference glass, named CEL2, had the following molar composition: 45% SiO_2_, 3% P_2_O_5_, 26% CaO, 7% MgO, 15% Na_2_O, 4% K_2_O. The glass was prepared by melting reagent-grade reactants in a platinum crucible at 1400 °C for 1 h. The molten glass was poured into water to obtain a frit that was subsequently ball-milled to obtain powders, which were later manually sieved. CEL2 glass powder was analyzed through differential thermal analysis (DTA, Netzsch STA 429, Selb, Germany), with heating rate of 10 °C/min up to 1000 °C in air, to evaluate its characteristic temperatures. For foaming experiments, only powders with a diameter below 75 µm were considered.

### 2.2. Preparation and Microstructural Characterization of Foams

To fabricate CEL2 glass-ceramic scaffolds for bone substitutions, fine CEL2 glass powders were mixed with an aqueous solution containing 1 M NaOH (reagent grade, Sigma-Aldrich, Gillingham, UK), for a solid loading of 58 and 60 wt %. The glass slurries were kept under low speed mechanical stirring (500 rpm) for 3h, for alkaline activation and partial gelation, and then cast in several polystyrene cylindrical moulds (60 mm diameter). After the addition of 4 wt % Triton X-100 (polyoxyethylene octyl phenyl ether—C_14_H_22_O(C_2_H_4_O)n, n = 9–10, Sigma-Aldrich, Gillingham, UK), consisting of a non-ionic surfactant that does not interfere with ceramic dispersions [23], the slurries were foamed by vigorous mechanical mixing (2000 rpm), for 5 min.

**Figure 1:**
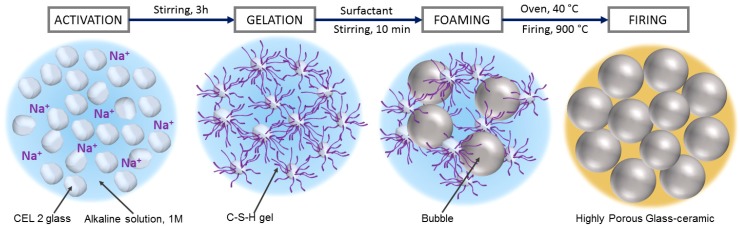
Processing scheme for the obtainment of CEL2 glass-ceramic foams by combination of alkali activation, gel casting and sintering.

The foamed slurries were later left at 40 °C for 24 h, in order to complete the gelation, before demolding. The obtained ‘green’ foams could be easily handled and placed in a muffle furnace, for the final sintering treatments, at 900–1000 °C for 1 h, followed by natural cooling. The thermal treatment comprised an intermediate step at 300 °C for 2 h to remove any absorbed water and organic residues, after a slow heating phase (2 °C/min); the subsequent heating up to the selected firing temperature was performed at 5 °C/min. Figure 1 describes the process used for manufacturing CEL2 glass-ceramic foams.

### 2.3. Characterization and Microstructural Investigation of CEL2 Foams

For understanding the thermal behavior, CEL2 glass powders and foamed gels were subjected to thermogravimetric analysis (TGA, STA409, Netzsch Gerätebau GmbH, Selb, Germany).

Fourier transform IR spectroscopy (FTIR, FTIR model 2000, Perkin Elmer Waltham, MA, USA) measurements were made for the dry CEL2 glass powder and for CEL2 glass foams, with a solid load of 60 wt %, in form of green foams and after heat treatment at 900 °C. FTIR analysis were conducted on solid discs (with 13 mm diameter), prepared by mixing powdered samples with KBr powder (2 mg dispersed in 200 mg of KBr). The FTIR spectra were collected in the 4000–400 cm^−1^ range.

The phase assemblages were performed by means of X-ray diffraction on powdered samples (XRD; Bruker D8 Advance, Bruker AXS GmbH, Karlsruhe, Germany), supported by data from PDF-2 database (ICDD-International Centre for Diffraction Data, Newtown Square, PA, USA) and Match! program package (Crystal Impact GbR, Bonn, Germany). X-ray diffraction analysis were performed using a scanning range 2θ from 10° to 70° with a scanning rate of 0.05°/step, and an analysis time of 2 s/step.

Nano-CT (SkyScan 1272, Bruker microCT, Kontich, Belgium) experiments were performed on CEL2 glass-ceramic foams with different solid loads, using a X-ray beam generated by a tungsten filament charged by a voltage of 70 kV and a current of 142 µA. Samples were analyzed over a rotation range of 180°, with a step of 0.02°, an exposure time of 3250 ms per projection and a pixel size of 5.37 µm. The so-collected shadow images have been first reconstructed using a filtered back-projection algorithm (NRecon, Bruker, Bruker microCT, Kontich, Belgium), then sample 3-D structures was obtained from the reconstructed images by means of a specific software (Bruker CTvox software, Mannheim, Germany). A volume of interest (VOI) was selected for each sample in order to analyze its structural parameters (Bruker CTan Software, Mannheim, Germany), after image binarization. In particular, for the analysis of pore interconnectivity the “shrink wrap” function has been applied in the 3D VOI. This function allows shrinking the outside boundary of the VOI through any openings whose connection size is larger than a threshold value (set in this work from 0 µm to 170 µm), identifying consequently the inaccessible regions of the scaffold comprised in the VOI. For each threshold value, the % interconnectivity has been calculated applying the following equation:

(1) $$\%\;Interconnectivity=\frac{\left(V-V_{shrink-wrap}\right)}{\left(V-V_{m}\right)}\times 100$$

where *V* is the total VOI volume, *V_shrink-wrap_* is the total volume after the application of the shrink-wrap function and *V_m_* is the scaffold volume comprised in the VOI.

Microstructural characterizations were performed by optical stereomicroscopy (AxioCam ERc 5 s Microscope Camera, Carl Zeiss Microscopy, Thornwood, NY, USA) and scanning electron microscopy (SEM, FEI Quanta 200 ESEM, FEI Company, Eindhoven, The Netherlands) equipped with energy dispersive spectroscopy (EDS).

The geometrical density (ρ_geom_) of CEL2 glass foams was evaluated by considering the mass to volume ratio. The apparent (ρ_app_) and the true (ρ_true_) density values were measured by using a helium pycnometer (AccuPyc 1330, Micromeritics, Norcross, GA, USA), operating on bulk or on finely crushed samples, respectively. The three density values were used to compute the amounts of total and open porosity.

The compressive strength (σ_c_) of CEL2 glass-ceramic foams was measured at room temperature, by means of an Instron 1121 UTM (Instron, Danvers, MA, USA) operating with a cross-head speed of 1 mm/min. Each data point represents the average value of 5 to 10 individual tests.

## 3. Results and discussion

**Figure 2:**
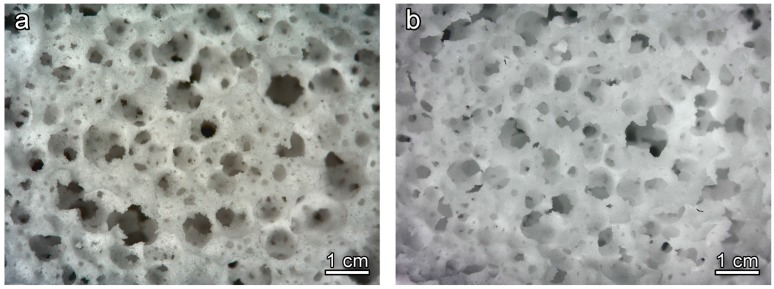
Microstructural and morphology details of CEL2 glass-ceramic foams, with 60 wt %, as solid load; (**a**) cured CEL2 green foam; (**b**) after firing at 900 °C.

Figure 2a clearly illustrates the great potential of the approach, despite its simplicity. Homogeneous and highly porous open-celled structures were achieved already in the ‘green’ state. As previously observed [21], the partial gelation of glass slurries, upon alkaline activation, leads to a marked pseudoplastic behavior, in analogy with what happens with more common ‘inorganic polymers’ (including geopolymers). Air bubbles, incorporated by intensive mechanical stirring (aided by the surfactant), at high shear rates and low viscosity, remain trapped when stirring stops, at low shear rate and high viscosity [24]. In the present investigation, there was an increase of 100–150% in the volume of slurries, passing from the alkali activation step to the hardened state.

The gelation of CEL2 glass was determined by the same compounds previously found after alkali activation of other CaO-rich glasses [21,22]. More precisely, CaO-rich glasses, upon alkali activation, are known to yield a ‘zeolite-like’ gel (like that formed in geopolymeric systems, based on the condensation of alumino-silicate hydrated oligomers, made available by complete alkali dissolution of alumino-silicate materials), but provide a condensation product that could be termed ‘tobermorite-like’ gel (calcium silicate hydrate, or C–S–H), given the analogy with the products of cement hydration [25].

**Figure 3:**
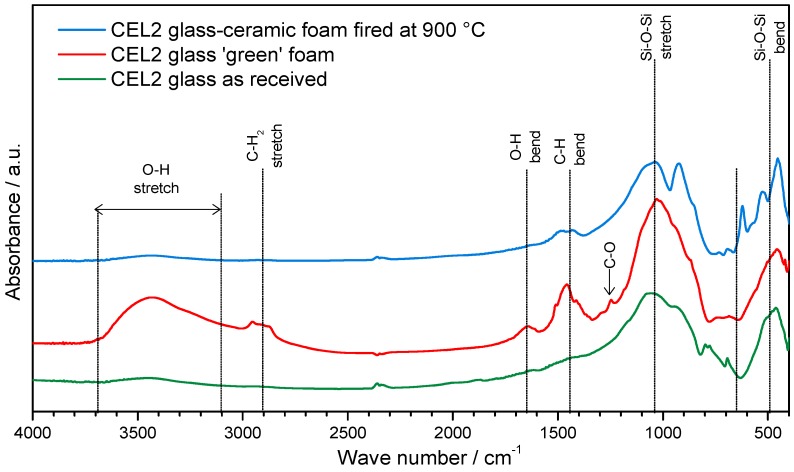
FTIR analysis of CEL2 glass, green CEL2 glass foams after alkaline activation and CEL2 glass-ceramic foam after firing.

The proof of C–S–H formation was given by FTIR analysis, as illustrated by Figure 3. In particular, the distinctive band associated to O–H stretching, in the 3200–3700 cm^−1^ range [21], along with a band at approximately 1650 cm^−1^, attributed to O–H bending, was clearly evident only in the FTIR spectrum of foams in the ‘green’ state, i.e., after gelation.

The bands from 1300 to 900 cm^−1^ and 800 to 450 cm^−1^, related to stretching and bending mode of Si-O-Si bonds [26,27], appeared wider and flattened, in the case of CEL2 green foam (compared to those of pure glass powders), as an effect of partial dissolution of the glass and formation of disordered hydrated gel.

Finally, further differences between green foams and starting glass concerned bands at 2950–2800 cm^−1^, 1500–1400 cm^−1^ and approximately 1250 cm^−1^. The first two bands were attributed to the IR absorption phenomena of the C–H bonds in the organic surfactant, whereas the third small band was attributed to the slight formation of carbonate compounds.

The FTIR spectrum of glass-ceramic foams was very similar to that of the starting glass, as an effect of the thermal instability of the hydrated compounds developed upon hardening. The only significant difference concerned the appearance of new bands at low wave number (at 1040, 925, 620, 530 and 450 cm^−1^), attributed to the formation of silicate crystalline phases.

**Figure 4:**
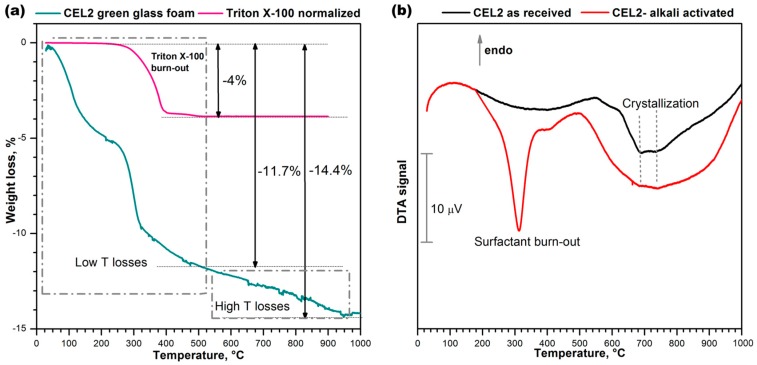
Thermal analysis of CEL2 glass before and after gel-casting; (**a**) Thermo-gravimetric plot of alkali-activated CEL2 glass and surfactant “Triton X-100”; (**b**) Differential thermal analysis of CEL2 glass powder and ‘green’ glass foam from alkali activation and direct foaming.

The thermal instability of the compounds developed upon hardening was confirmed by the thermogravimetric analysis (TGA), shown in Figure 4a. Weight losses appeared at several stages, as a consequence of the overlapping of distinct phenomena, occurring below 500 °C (low T losses) and above 500 °C, up to about 900 °C (high T losses). The low temperature losses comprise a contribution from the thermal decomposition of the surfactant, illustrated by the same Figure 4a. The contribution was significant, but the low temperature losses (~11.7%) were far above the overall content of the same additive (for comparison purposes, Figure 4a reports the weight losses of Triton X-100 normalized by the effective amount of additive employed, i.e., 4%). Like in previous cases [21,22], we cannot exclude a remarkable contribution to the low temperature losses of physically adsorbed water (below 200 °C) and decomposition of hydrated compounds.

The occurrence of high temperature losses is consistent with the same nature of C–S–H gels. In fact, C–S–H compounds are actually known to release water even above 500 °C [28].

The differential thermal analysis (DTA) plots in Figure 4b, for the starting glass and for a foam sample just after low temperature hardening, are particularly interesting for the two exothermic peaks. The low temperature strong peak, visible only in the plot for the green foam, undoubtedly corresponds to the burn-out of the surfactant, considering the exact match (at 300 °C) with the onset of the relative thermal loss (see upper plot in Figure 4a). The broader exothermic peaks at higher temperatures are significant for the differences between starting glass and green foam: in the as-received condition, CEL2 glass presented a broad ‘exothermic band’ that could be attributed to the overlapping of at least two crystallization peaks, at 700 and 750 °C; in the activated condition, the band became even wider and positioned at lower temperatures. This effect had not been detected with the alkali activation of previously investigated CaO–MgO–SiO_2_ glass [22]. In our opinion this could be justified on the basis of the mixing of two distinct glass phases, consisting of material from the decomposition of the surface gels and undissolved glass. In particular, the alkali enrichment of surface gels surrounding glass powders likely lowered the glass transition temperature (Tg), promoting the ionic inter-diffusion and the crystallization of CEL2 glass (e.g., by reducing the activation energy for crystal growth [29]). The endothermic effect centered at about 500 °C was attributed to dehydration of the green foam.

The promotion of the crystallization had a remarkable impact on the microstructure after firing. As shown by Figure 2b the open-celled structure was confirmed after firing at 900 °C: the increase of apparent viscosity, operated by rigid crystal inclusion, evidently prevented any viscous collapse.

**Figure 5:**
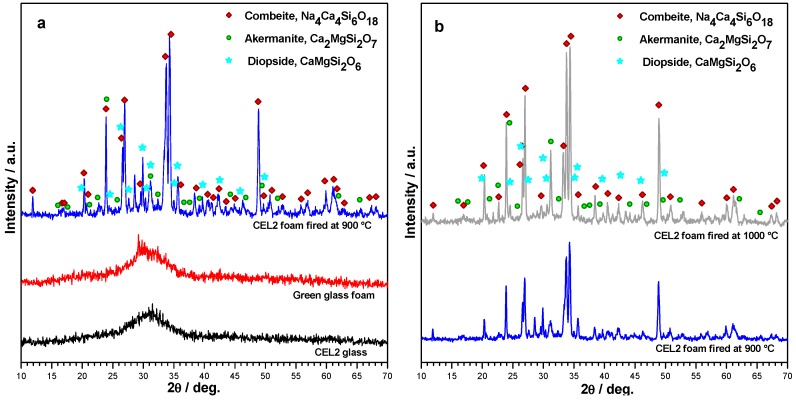
X-ray diffraction analysis of CEL2 glass-based materials: (**a**) evolution from as received state to alkali-activated and sinter-crystallized state; (**b**) comparison between firing at 900 and 1000 °C (foamed samples, from 60% CEL2 solid loading).

The X-ray diffraction patterns reported in Figure 5 provide an overview of the evolution of crystalline phases in the processing of CEL2 glass-ceramic foams. The XRD pattern of the alkaline-activated material provided further evidence of gel formation, after foaming and drying of the glass, due to the slight 2θ displacement the amorphous halo (Figure 5a). The 2θ shift for the green glass foams, in fact, is consistent with the incorporation of network modifiers [30]. After firing at 900 °C, the typical crystalline phases of bioactive CEL2 glass-ceramic, such as combeite (Na₄Ca₄Si₆O₁₈, PDF#79-1089) and akermanite (Ca₂MgSi₂O₇, PDF#83-1815) [13], appeared. Interestingly, another Ca-Mg silicate (diopside, CaMgSi₂O₆, PDF#75-1092) formed as additional phase; to our opinion, this could be due the decomposition of the hydrated silicate gels formed upon activation. The crystallization degree (inferable from the intensity of diffraction lines) increased passing from 900 to 1000 °C (see Figure 5b); in addition, there was a reduction of diopside, with increase of akermanite. In other words, the firing at 1000 °C made the phase assemblage of the present foams more similar to that previously reported [13]. The formation of Ca–Mg silicate crystal phases (akermanite or diopside) is very promising, due to the distinctive combination of remarkable mechanical strength with excellent bioactivity as well as controlled dissolution rate [31,32].

Table 1 reports the physical and mechanical properties of CEL2 glass-ceramic foams after firing. It can be noticed that samples were produced according to several processing variants, aimed at exploring different viscosity conditions before firing and during firing. In fact, both steps could be interpreted according to the mechanics of suspensions, in which the apparent viscosity depends on the combination of the viscosity of the medium and the amount of suspended, rigid inclusions. An increase of the solid loading, from 58 to 60 wt %, was expected to cause an increase of the viscosity of slurries simply on the basis of the amount of inclusions, reducing the expansion upon intensive mechanical stirring. An increase of the firing temperature, from 900 to 1000 °C, on one hand, was expected to reduce the viscosity of the residual glass phase, i.e., of the medium in which crystal inclusions were suspended, enhancing the densification by removal of smaller pores; on the other hand, the increased crystallization degree (Figure 5b) could determine a substantial reinforcement of the solid phase. The data reported in Table 1 confirm the expected ‘tuning’ of porosity operating on solid loading and/or on firing temperature. With a compressive strength ranging from 2.5 ± 0.4 to 15.5 ± 1.7 MPa, the developed foams compare well with the values for cancellous bone (2–12 MPa) [33,34].

It should be noted that the lightest foam, processed at low solid loading and low firing temperature and exhibiting the lowest strength, could not be considered as ‘weak’. The crushing strength of cellular solid, σ_c_, according to the well-recognized Gibson-Ashby (GA) model [35], actually depends on the relative density (ratio between bulk and true densities, ρ_rel_), as follows:

σ_c_ ≈ σ_bend_ × f(Φ,ρ_rel_) = σ_bend_ × [C × (Φ·ρ_rel_)^3/2^ + (1 − Φ) × ρ_rel_]

where σ_bend_ is the bending strength of the solid phase and f is a ‘structural function’, depending on the relative density (ρ_rel_, the ratio between the bulk density of the foams and the true density, i.e., the density of the solid phase) and its distribution (open or closed porosity). The quantity (1 − Φ) expresses the fraction of solid positioned at the cell faces; C is a dimensionless calibration constant (∼0.2). For the lightest foam, the amount of closed porosity was so limited that the linear contribution in the structural function could be neglected (Φ ∼ 1); the observed compressive strength could be correlated, given the low relative density, to a bending strength in the order of 150 MPa, typical for glass-ceramics [36].

GA models are actually based on the assemblage of mono- and bi-dimensional elements, neglecting the stress concentrations associated with the junctions of the same elements [37] and the structural in-homogeneity, e.g., for foams at an intermediate stage between perfectly closed cell foams and lattice-structured foams, with cell walls featuring holes at the centre [38]. In the hypothesis of poorly-collaborating cell walls, high Φ values could be attributed also to foams with limited open porosity. With Φ values from 0.9 to 1, the sample sintered at 900 °C, starting from 60 wt % solid loading, features a bending strength of 70 MPa–120 MPa, also in line with the strength values of glass-ceramics.

**Figure 6:**
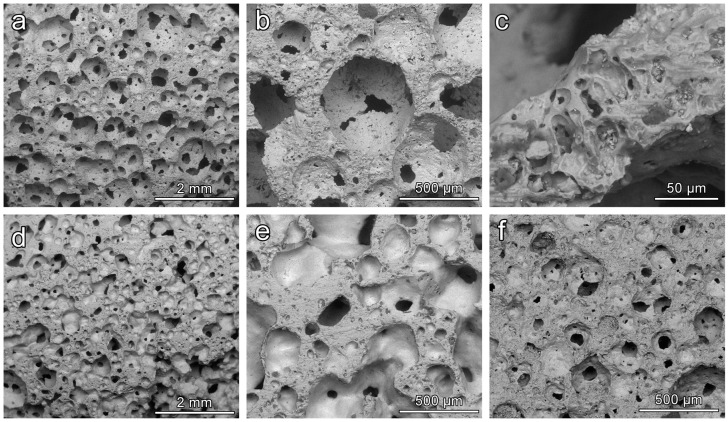
Examples of CEL2 glass-ceramic foams with different solid content (wt %) and after firing at 900–1000 °C for 1 h; (**a**–**c**) foams with 58 wt % solid loading fired at 900 °C; (**d**,**e**) for foams with 60 wt % solid loading fired at 900 °C; (**f**) foams with 58 wt % solid loading fired at 1000 °C.

Figure 6 reports a selection of microstructural details of CEL2 glass-ceramics, collected by means of SEM, obtained from slurries with 58 and 60 wt % solid content and fired at different temperatures. In all cases it is possible to observe the presence of quite homogeneously distributed interconnected cells. We can also roughly note a sort of ‘synergy’ between solid content and firing temperature. A low firing solid loading and a low firing temperature led to foams (Figure 6a) with pronounced interconnectivity, i.e., with many openings between adjacent cells (Figure 6b). The struts were by themselves highly porous (see Figure 6c), promoting the cell attachment and the impregnation of fluids. An increase of solid loading (Figure 6d,e) or firing temperature (Figure 6f) determined the formation of some solid membranes between adjacent cells: the strength enhancement can be understood on the basis of an increased amount of solid positioned at the cell faces (linear term in the structural function ruling the impact of relative density on compressive strength).

**Figure 7:**
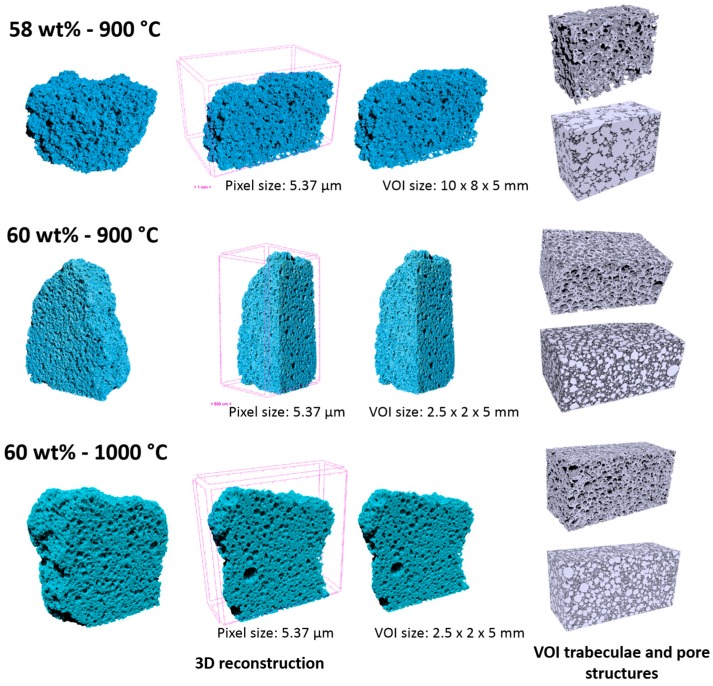
Nano-CT analysis of CEL2 glass-ceramics foams with different solid contents and fired using different heat-treatments.

The pore distribution and interconnectivity were further analyzed using nano-CT, which yielded a three-dimensional representation of foams, as illustrated by Figure 7. The analysis was actually restricted to the foams fired at low temperature and to that exhibiting the highest strength (60% solid loading, fired at 1000 °C), and was specifically intended to verify the suitability of the developed foams in terms of morphological requirements for tissue engineering. In fact, an ideal porous scaffold should combine bioactive properties with a favorable structure, i.e., the interconnections between adjacent cells should have a diameter exceeding 100 µm, to allow effective tissue ingrowth and eventually vascularization (required for complete bone regeneration) [39].

The lightest foam (58% solid loading, fired at 900 °C) effectively exhibited a very abundant porosity: the light grey volume in the 3D reconstruction images (top part of Figure 7), associated to the porosity, is largely dominant and large connections are evident. This condition actually forced us to refer to a bigger volume of interest (10 mm × 8 mm × 5 mm), compared to other systems (evaluated on a VOI of 2.5 mm × 2 mm × 5 mm).

**Figure 8:**
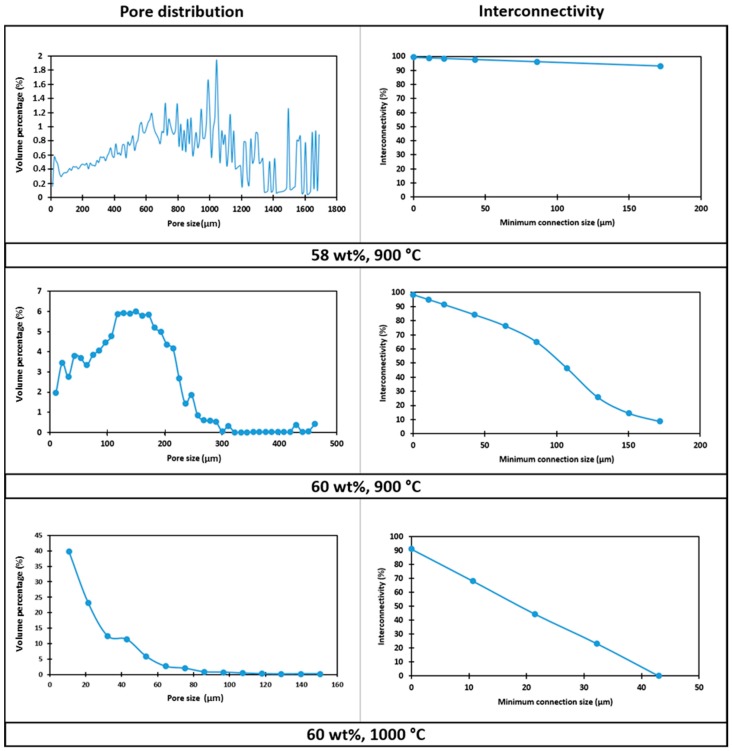
Pore size distribution and interconnectivity of CEL2 glass-ceramic foams, produced by different solid contents, after heat-treatment.

The large variability of pore size (mostly between 300 μm and 1.5 mm, see Figure 8) could be attributed to the low viscosity of the starting slurry: big interconnected pores, surrounded by smaller ones, were likely formed by coalescence of adjacent bubbles in the shaping step; the structure remained ‘frozen’ as an effect of limited viscous flow, upon low temperature firing.

The foam developed with a 60 wt % solid loading in the starting slurry and still sintered at 900 °C, despite the appearance of solid membranes (Figure 6e), maintained a very good interconnectivity. From Figure 7 (central part), we can note that the porosity was particularly homogeneous. From the analysis of reconstructed 3D images (Figure 8), the porosity was in a much more limited range, between 5 and 300 μm, with mean diameter of 170 μm; the shrink-wrap function, used to calculate the interconnection, yielded pores above the previously mentioned threshold of 100 μm.

The nominally strongest foam (60% solid loading, fired at 1000 °C), although still very porous, did not fulfil the requirements for bone tissue engineering, since most of the pores and interconnections were below 50 μm (see Figure 8). The increase of firing temperature, as a consequence, cannot be considered as a valid ‘tuning’ parameter for the chosen application.

We can conclude that foams sintered at 900 °C may constitute a valid reference for future bone tissue engineering experiments. The absolute strength (above 5 MPa, for 60% solid loading) and strength-to-density ratio (especially for 58% solid loading), combined with the optimum pore distribution, make them good candidates for bone tissue regenerations.

## 4. Conclusions

We may conclude that:

- Highly porous CEL2 glass-ceramics can be easily manufactured by ‘inorganic gel-casting’, followed by sintering with sinter-crystallization; the crystallization limits the viscous flow, so that the microstructure in the green state is substantially maintained upon firing up to 900–1000 °C;
- The overall foaming process (mechanical stirring of alkali activated suspensions—with the help of a surfactant, drying, firing with sinter-crystallization) has a great potential for the production of ‘hierarchically porous’ foams; the microstructure can be tuned operating on simple processing parameters such solid load, in suspensions, and firing conditions (e.g., heating rate);
- CEL2 glass-ceramic foams were fabricated, in selected conditions, with very uniform pore size (mean diameter of 170 μm) and good interconnectivity (well-defined openings are visible between adjacent cells).

## Figures and Tables

**Table 1:** Physico-mechanical properties of CEL2 glass-ceramic foams produced by different solid contents (TP = total porosity, OP = open porosity)

Solid Load (wt. %)	T (°C)	ρ_geom_ (g/cm^3^)	ρ_app_ (g/cm^3^)	ρ_true_ (g/cm^3^)	Relative Density, ρ_rel_	TP (vol %)	OP (vol %)	σ_c_ (MPa)
58	900	0.58 ± 0.03	2.38 ± 0.01	3.06 ± 0.01	0.19	81 ± 5	75 ± 5	2.5 ± 0.4
	1000	0.92 ± 0.07	2.05 ± 0.09	3.13 ± 0.02	0.30	70 ± 8	55 ± 1	3.9 ± 0.5
60	900	1.12 ± 0.02	2.49 ± 0.09	3.05 ± 0.01	0.37	63 ± 2	55 ± 5	5.5 ± 0.2
	1000	1.16 ± 0.08	2.52 ± 0.04	3.16 ± 0.01	0.37	63 ± 7	52 ± 8	15.5 ± 1.7

## References

[1] Tampieri A., Celotti G., Landi E. (2005). From biomimetic apatites to biologically inspired composites. Anal. Bioanal. Chem..

[2] Hutmacher D.W. (2000). Scaffolds in tissue engineering bone and cartilage. Biomaterials.

[3] Ramakrishna S., Meyer J., Wintermantel E., Leong K.W. (2001). Biomedical applications of polymer-composite materials: A review. Comput. Sci. Technol..

[4] Jones J.R. (2013). Review of bioactive glass: From Hench to hybrids. Acta Biomater..

[5] Hench L.L. (1991). Bioceramics: From Concept to Clinic. J. Am. Ceram. Soc..

[6] Kokubo T., Takadama H. (2006). How useful is SBF in predicting in vivo bone bioactivity?. Biomaterials.

[7] Rabiee S.M., Nazparva N., Azizian M., Vashaee D., Tayebi L. (2015). Effect of ion substitution on properties of bioactive glasses: A review. Ceram. Int..

[8] Wren A.W. (2016). 45S5 Bioglass Based Scaffolds for Skeletal Repair: Biocompatible Glasses. Adv. Struct. Mater..

[9] Kaysinger K.K., Ramp W.K. (1998). Extracellular pH modulates the activity of cultured human osteoblasts. J. Cell. Biochem..

[10] El-Ghannam A., Ducheyne P., Shapiro I.M. (1997). Formation of surface reaction products on bioactive glass and their effects on the expression of the osteoblastic phenotype and the deposition of mineralized extracellular matrix. Biomaterials.

[11] Brandao-Burch A., Utting J.C., Orriss I.R., Arnett T.R. (2005). Acidosis inhibits bone formation by osteoblasts in vitro by preventing mineralization. Calcif. Tissue Int..

[12] Vitale-Brovarone C., Verne´ E., Robiglio L., Martinasso G., Canuto R.A., Muzio G. (2008). Biocompatible glass-ceramic materials for bone substitution. J. Mater. Sci. Mater. Med..

[13] Vitale-Brovarone C., Baino F., Verne E. (2009). High strength bioactive glass-ceramic scaffolds for bone regeneration. J. Mater. Sci. Mater. Med..

[14] Porter N.L., Pilliar R.M., Grynpas M.D. (2001). A review of materials, fabrication methods, and strategies used to enhance bone regeneration in engineered bone tissues. J. Biomed. Mater. Res..

[15] Vitale-Brovarone C., Verné E., Robiglio L., Appendino P., Bassi F., Martinasso G., Muzio G., Canuto R. (2007). Development of glass-ceramic scaffolds for bone tissue engineering: Characterisation, proliferation of human osteoblasts and nodule formation. Acta Biomater..

[16] Vitale-Brovarone C., Miola M., Balagna C., Verné E. (2008). 3D-glass–ceramic scaffolds with antibacterial properties for bone grafting. Chem. Eng. J..

[17] Lyckfeldt O., Ferreira J.M. (1998). Processing of porous ceramics by starch consolidation. J. Eur. Ceram. Soc..

[18] Vitale-Brovarone C., Di Nunzio S., Bretcanu O., Vernné E. (2004). Macroporous glass-ceramic materials with bioactive properties. J. Mater. Sci. Mater. Med..

[19] Vitale-Brovarone C., Verné E., Appendino P. (2006). Macroporous bioactive glass-ceramic scaffolds for tissue engineering. J. Mater. Sci. Mater. Med..

[20] Baino F., Ferraris M., Bretcanu O., Verné E., Vitale-Brovarone C. (2013). Optimization of composition, structure and mechanical strength of bioactive 3-D glass-ceramic scaffolds for bone substitution. J. Appl. Biomater..

[21] Rincón A., Giacomello G., Pasetto M., Bernardo E. (2017). Novel ‘inorganic gel casting’ process for the manufacturing of glass foams. J. Eur. Ceram. Soc..

[22] Elsayed H., Rincón Romero A., Ferroni L., Gardin C., Zavan B., Bernardo E. (2017). Bioactive Glass-Ceramic Scaffolds from Novel ‘Inorganic Gel Casting’ and Sinter-Crystallization. Materials.

[23] Wang X., Ruan J., Chen Q. (2009). Effects of surfactants on the microstructure of porous ceramic scaffolds fabricated by foaming for bone tissue engineering. Mater. Res. Bull..

[24] Rincon Romero A., Elsayed H., Bernardo E. (2018). Highly porous mullite ceramics from engineered alkali activated suspensions. J. Am. Ceram. Soc..

[25] Provis J.L. (2014). Geopolymers and other alkali activated materials: Why, how, and what?. Mater. Struct..

[26] Shah A.T., Batool M., Chaudhry A.A., Iqbal F., Javaid A., Zahid S., Ilyas K., Bin Qasim S., Khan A.F., Khan A.S. (2016). Effect of calcium hydroxide on mechanical strength and biological properties of bioactive glass. J. Mech. Behav. Biomed. Mater..

[27] Chakradhar R.S., Nagabhushana B.M., Chandrappa G.T., Ramesh K.P., Rao J.L. (2006). Solution combustion derived nanocrystalline macroporous wollastonite ceramics. Mater. Chem. Phys..

[28] Zhang Q., Ye G. (2012). Dehydration kinetics of Portland cement paste at high temperature. J. Therm. Anal. Calorim..

[29] Watanabe T., Hashimoto H., Hayashi M., Nagata K. (2008). Effect of Alkali Oxides on Crystallization in CaO–SiO2–CaF2 Glasses. ISIJ Int..

[30] Hemmings R., Berry E. (1987). On the glass in coal fly ashes: Recent advances. MRS Proceedings.

[31] Wu C., Chang J. (2013). A review of bioactive silicate ceramic. Biomed. Mater..

[32] Mohammadi H., Sepantafar M., Ostadrahimi A. (2015). The Role of Bioinorganics in Improving the Mechanical Properties of Silicate Ceramics as Bone Regenerative Materials. J. Ceram. Sci. Tech..

[33] Thompson I.D., Hench L.L. (1998). Mechanical Properties of Bioactive Glasses, Glass-Ceramics and Composites. Proc. Inst. Mech. Eng. Part H. J. Eng. Med..

[34] Rahaman M.N., Liu X., Huang T.S., Narayan R., Colombo P. (2009). Bioactive glass scaffolds for the repair of load-bearing bones. Advances in Bioceramics and Porous Ceramics.

[35] Gibson L.J., Ashby M.F. (1999). Cellular Solids, Structure and Properties.

[36] Höland W., Beall G. (2002). Glass-Ceramic Technology.

[37] Rice R. (1998). Porosity of Ceramic.

[38] Rice R., Scheffler M., Colombo P. (2005). Mechanical Properties. Cellular Ceramics: Structure, Manufacturing, Properties and Applications.

[39] Hulbert S.F., Morrison S.J., Klawitter J.J. (1972). Tissue reaction to three ceramics of porous and non-porous structures. J. Biomed. Mater. Res..

